# Drivers or Drifters? The “Who” and “Why” of Leader Role Occupancy—A Mixed-Method Study

**DOI:** 10.3389/fpsyg.2021.573924

**Published:** 2021-03-04

**Authors:** Elina Auvinen, Mari Huhtala, Johanna Rantanen, Taru Feldt

**Affiliations:** Department of Psychology, University of Jyväskylä, Jyväskylä, Finland

**Keywords:** leader emergence, leader role occupancy, motivation to lead, sustainable career, occupational well-being, person-career fit

## Abstract

This study investigated the reasons that leaders have given for their leader role occupancy. By using a mixed-method approach and large leader data, we aimed to provide a more nuanced picture of how leader positions are occupied in real life. We examined how individual leadership motivation may associate with other reasons for leader role occupancy. In addition, we aimed to integrate the different reasons behind leader role occupancy into the framework of sustainable leader careers and its two indicators: leader’s health (occupational well-being) and performance (measured indirectly as followers’ occupational well-being). The survey data consisted of 1,031 leaders from various sectors of working life. Qualitative analysis revealed that leaders mention various factors behind their leader role occupancy, resulting 26 themes. After inductive investigation of the data, theory-driven analysis focused on the sustainable career components (person, context, time) and agency vs. non-agency. Qualitative data was quantitized based on the theory-driven categories for statistical analysis. Based on the these analysis, we found out that only Affective-Identity MTL predicted all of the studied reasons behind leader role occupancy, whereas the other motivation types (Non-calculative MTL and Social-Normative MTL) did not. All of the reasons for leader role occupancy except non-agentic ones were related to both leaders’ own and their followers’ occupational well-being. Leaders with more person-related and agentic reasons for leader role occupancy experienced better occupational well-being. Person- and context-related and agentic reasons behind leader role occupancy associated also with followers’ occupational well-being, but the associations differed from those of leaders’ well-being: person-related and agentic reasons associated with followers’ exhaustion, but this association was not found among leaders. Our study provided important information for practitioners in the field of human resources and development, as it has shown that if the reasons for leader role occupancy mainly reflect circumstances or other non-person-related reasons, the experienced occupational well-being and person-career fit may remain weak. It is necessary to try to support the leadership motivation for those leaders, or to shape the job description in such a way that it can also offer the experiences of meaningfulness from aspects other than self-realization through a managerial role.

## Introduction

Despite the long research tradition on leadership, empirical and scientific research has not led to a conclusive understanding of how leadership emergence actually takes place among individuals who are acting in complex environments, such as employees and managers working in different organizations. Most of the research on leadership emergence is based on artificial, situation- and participant-specific group simulations, especially leaderless group discussions (e.g., Ensari et al., 2011). For a thorough exploration of who emerges as a leader, other techniques or perspectives in addition to leaderless group discussions are needed.

Leader emergence is not a straightforward, static phenomenon, and this makes it more difficult to capture and examine. As Acton et al. (2019) summed it up, “leadership emergence is more than a trait, an exchange, or a symbol—leadership emerges through dynamic interactions (Lichtenstein et al., 2006) at multiple levels” (p. 146). Thus, the *process* perspective of leader emergence deserves more attention than it has previously been given. The studies conducted so far have treated concepts of leadership emergence and leader role occupancy either as a predictor or as an outcome variable (Tuncdogan et al., 2017; Zaccaro et al., 2018). However, it is clear that approaching an emergent, process-like phenomenon (the question of who will eventually occupy a leader position) by reducing it to a single factor or one end result is likely to lead to the omission of relevant aspects. In addition to understanding the process of leader emergence more systematically, it is important to investigate how the leader emergence process associates with leader careers, and how these careers unfold.

Recently, the overemphasis on individual reasons and the under-emphasis on the situational or contextual factors of leader emergence has received attention in the literature (Hanna et al., 2021). Hanna et al. (2021) also called for the need for conceptual clarification and sound ways to operationalize leader (ship) emergence. As a way to address these shortcomings in the literature and to provide a more realistic and balanced view of the leadership emergence process, we adopted a mixed-methods approach using both qualitative and quantitative methods to investigate the reasons the leaders gave for occupying their leadership role as a starting point for our analysis. Tashakkori and Creswell (2007) defined mixed methods research as a process “in which the investigator collects and analyzes data, integrates the findings, and draws inferences using both qualitative and quantitative approaches or methods in a single study or a program of inquiry” (p. 4). We used a merged concurrent nested approach (Castro et al., 2010; Fetters et al., 2013) to be able to analyze the real-life experiences of leaders with accurately measured constructs (Castro et al., 2010). More specifically, we examined the leaders’ qualitative descriptions about their reasons for occupying their leadership role and, after theory-driven classification, we quantitized (Teddlie and Tashakkori, 2009) their descriptions to investigate their associations to sustainable leader career components and personal leadership motivation. Integrating different reasons behind leader role occupancy into the wider framework of sustainable leader careers (De Vos et al., 2020) is, to the best of our knowledge, a novel perspective in this research area. This integration of qualitative and quantitative data through data conversion enabled us to capture a richer and more detailed picture of why, how, and under what circumstances leader positions are occupied in real life and how the reasons the leaders provided are associated with the sustainability of their careers as a leader and their personal leadership motivation.

How a person’s career actually unfolds over time is determined by individual choices made at a specific moment in time and affected by various factors, such as social or organizational context (Rudolph et al., 2019; Urbanaviciute et al., 2019; van der Horst and Klehe, 2019). Leader role occupancy can be viewed as one kind of choice, and it (and more broadly, the whole process of leader emergence) may therefore act as a stimulus to a further career as a leader. Thus, in addition to provide a nuanced picture of how leader positions are occupied, our specific interest is especially to investigate the *sustainability* of leader careers (De Vos et al., 2020) in relation to the reasons that have affected leader role occupancy. As indicators of career sustainability, a leader’s health (occupational well-being) and performance (reflected in followers’ occupational well-being) are studied. We begin by introducing the individual factors, specifically personal leadership motivation, that associate with leader emergence and how leader emergence relates to sustainable leader careers and its focal indicators.

### Motivation to Lead as a Personal Factor Behind Leader Emergence

Motivation to Lead (MTL) provides one perspective to explain leader emergence (Chan and Drasgow, 2001). Chan and Drasgow (2001) have stated that MTL is “an individual-differences construct that affects a leader’s or leader-to-be’s decisions to assume leadership training, roles, and responsibilities” (p. 482). Thus, it is a central concept of leader development, highlighting its process-like, dynamic nature. MTL is a multidimensional concept that consists of three distinct but related dimensions with different antecedents and related outcomes (Chan and Drasgow, 2001; for a meta-analysis, see Badura et al., 2020). *Affective-Identity MTL* refers to positive valence toward leadership and leading others and is considered the most intrinsic motivational dimension of leadership motivation. Those with high Affective-Identity MTL usually consider themselves natural born leaders. *Social-Normative MTL*, as a more extrinsic motivational component, is based on social norms: an individual with high Social-Normative MTL might lead out of a sense of duty or responsibility, or because they consider leader status to be normatively valued. Lastly, *Non-calculative MTL* refers to positive perceptions of leadership roles and formal positions, regardless of their potential costs or negative consequences (Badura et al., 2020). Because those with high Non-calculative MTL are likely to lead out of a general willingness, without weighing the possible costs and benefits related to leading others (Chan and Drasgow, 2001; Porter et al., 2019). Non-calculative MTL can be considered a “selfless” aspect of leadership motivation.

Some earlier studies have investigated the role of MTL in the leader emergence process (Hendricks and Payne, 2007; Hong et al., 2011; Oh, 2012; Luria and Berson, 2013; Mohan and Carter, 2019) using individual MTL as a predictor variable, mostly in cross-sectional settings, but the perspective of leader careers has not received much research attention in the MTL literature. Also, the existing research has failed to establish how an individual’s MTL associates with other factors that can affect leader role occupancy in complex environments, such as unanticipated organizational restructuring or sudden needs for personnel changes. In the original model by Chan and Drasgow (2001), only limited attention was given to the contextual factors that may shape and affect leader role occupancy in practice, in addition to MTL. Not only individual motivational factors, but also situational triggers or events in an organization may lead to one taking up the leader role.

From the broader career perspective, leader role occupancy at a certain time may (or may not) associate with future career decisions, when an individual weighs up future possibilities of pursuing leader roles. So far as we know, previous studies have not investigated MTL or reasons for leader role occupancy from a perspective that would capture future leader-career orientations. This gap in the literature needs to be addressed, as evidence shows that pursuing leader positions is not the most important career goal of the majority of students or employees (Chudzikowski, 2012; Sutela and Lehto, 2014; Torres, 2014; Crowley-Henry et al., 2019). If we are going to lack sufficient candidates for leader positions in the future, we need more information on how to make careers more lasting and how to support the construction of a sustainable career for those leaders who already occupy the position. To extend research on MTL and integrate that research into research on leader careers, we examine how individual factors, particularly individual leadership motivation, may associate with other reasons for leader role occupancy. In integrating these different perspectives in this study, we apply the conceptual model of sustainable careers (De Vos et al., 2020).

### Sustainable Leader Career as an Outcome of Leader Emergence

The conceptual model of sustainable careers (De Vos et al., 2020) explains how careers unfold in the interplay of three dimensions: the individual, context, and time (Chudzikowski et al., 2019; De Vos et al., 2020). The individual is seen as an agentic career actor, whose career possibilities are likely to be influenced by and to interact with his or her particular context (e.g., occupation, work group, organization) and time (e.g., career stage). The conceptual model of sustainable careers includes four central concepts: agency, meaning, proactivity, and adaptation (De Vos et al., 2020). Constructing a sustainable career is a dynamic process in which the interrelationship between these four focal concepts is manifested as person-career fit. In order to create and retain a good person-career fit, the individual as an agentic subject both proactively shapes his or her environment and, on the other hand, adapts to external forces. From the perspective of person-career fit, the importance of meaning cannot be overemphasized, as knowledge of one’s personal values and needs generates experience of what one understands to be meaningful work, and provides important knowledge for one’s further career decisions (De Vos et al., 2020).

Thus far, career theories have emphasized the role of individual agency in shaping a career from the vocational perspective, without paying much attention to organizational and institutional perspectives, which presume that also organizations and wider institutional forces affect individual careers (Inkson et al., 2012). In seeking to develop an understanding of how careers unfold, we need to apply the ideas of systems thinking to acknowledge the role of various factors affecting the career unfolding process. The sustainable careers framework acknowledges that individual agency and proactivity are likely to be affected by contextual demands and resources, and in addition to the individual’s own active endeavors, it also highlights adaptation and adjustment to environmental factors (De Vos et al., 2020). From the perspective of leader emergence and career continuity, we are interested in investigating whether individuals deliberately drive their way toward leader roles as active agents, or if they drift toward these roles under the influence of external forces. In addition, we examine if these processes of “driving” vs. “drifting” are associated with the focal indicators of sustainable careers, to which we will now turn.

### Indicators of Sustainable Leader Careers: Leader’s Health and Productivity

According to De Vos et al. (2020), the sustainability of a career can be assessed through three indicators: *happiness, health*, and *productivity.* Happiness refers to one’s personal satisfaction with one’s career and subjective career success, health is associated with both physical and psychological health and well-being, and productivity refers to performance in one’s current job, and the fit between the career and the organization’s needs for human capital. These three indicators of sustainable careers reflect the dynamic person-career fit and an individual’s success in adapting and/or proactively shaping contexts and dealing with environmental influences (De Vos et al., 2020). In this study, we focus on two of these career sustainability indicators, a leader’s *health* and *productivity*, the second of which was not measured directly but was inferred indirectly from leader performance.

As a *health* indicator, we examined leader’s occupational well-being, namely burnout and work engagement. Burnout develops in response to chronic job-related stressors, which result in experiences of emotional exhaustion (feelings of strain and fatigue), cynicism (a distal attitude toward one’s work or colleagues and a general loss of interest in one’s work), and reduced professional efficacy (feelings of incompetence at work) (Maslach and Jackson, 1981; Maslach et al., 2001). Work engagement is a three-dimensional concept of positive well-being at work, which is described as having high mental energy while working (vigor), a sense of significance, pride and inspiration (dedication) and immersion in one’s work (absorption) (Schaufeli et al., 2002, 2006). Burnout and work engagement are both known to be associated with career-relevant outcomes, such as organizational commitment, but each one in its own way: burnout is negatively associated with commitment, whereas work engagement increases commitment to one’s organization (Hakanen et al., 2008; Kim et al., 2013). In addition, burnout is associated with a growing intention of leaving the profession (Reinardy, 2011).

From the perspective of *productivity* and one of its indicators, performance, we are interested on how a leader performs his or her leadership-related duties and how this is manifested in the followers’ occupational well-being. We argue that this is a central viewpoint because leaders are influential figures in organizations and their performance and behaviors are relevant to their employees’ well-being and to the organization as a whole (e.g., Skakon et al., 2010; Ashford et al., 2018). Although performance can be assessed from various perspectives, we consider followers’ occupational well-being (low burnout, high work engagement) to be an important output of leadership because, in general, occupational well-being is an important indication of sustainable careers. Moreover, occupational well-being is one of the values that is essential to integrate into the leadership-related debate alongside the hard performance figures that are measured in money or profit.

We propose that the link from the leader’s performance to the occupational well-being of followers could be via the leader’s motivational resources, which may affect actual performance as a leader and various leadership behaviors. How well a leader performs his/her role-related duties in the workplace may be manifested in several ways: for example, in the quality of social relationships, such as the quality of the leader-member exchange (LMX) (Graen and Uhl-Bien, 1995), and in the leader’s transformational leadership skills, that is, his/her capacity to inspire, provide a clear vision, initiate structure, and support his/her followers (Bass, 1999).

The effort that leader puts into the relevant performance and leadership related behaviors might be dependent of leader’s motivational resources (Auvinen et al., 2020). According to the Conservation of Resources Theory (Hobfoll, 1998, 2001, 2011) and its principles concerning resource loss, if a leader has low (motivational) resources for leading others, these scarce resources have to be actively defended to avoid progressive resource loss. Defending initially scant resources is energy-consuming and it may result in the leader putting less effort into his or her work, that is, into leadership-related duties. There is some cross-sectional evidence that the leaders that had low or inadequate motivational resources for leadership reported more burnout symptoms and less work engagement (Auvinen et al., 2020), thus highlighting that those resources have a significant impact on the well-being of leaders. One meta-analytical review (Harms et al., 2017) examined leader stress as an antecedent of leader behaviors; they found preliminary support for the negative relationship between leader burnout and self-reported transformational leadership behaviors. This finding is also in line with the Conservation of Resources Theory (Hobfoll, 1998, 2001).

Despite the ambiguity concerning the actual moderating mechanism, the link from leader behaviors or leadership style to followers’ well-being has been strongly supported by previous research. According to a systematic literature review, positive leader behaviors and transformational leadership style were positively associated with employee affective well-being and low stress; the opposite was found for negative leader behaviors (Skakon et al., 2010). One cross-sectional study (Auvinen et al., 2020) showed that the leader’s motivational resources for leadership was associated with the followers’ assessment of their leader’s people- and task-oriented leadership behaviors and LMX quality: when leaders had low motivational resources, they received inferior ratings from their followers for their leadership behaviors and LMX quality. There is also meta-analytical support for leader behaviors as the cause of follower well-being. For example, Harms et al. (2017) found strong support for the association between transformational leadership style and high LMX quality and lower levels of follower burnout and stress. Specifically, poor LMX showed stronger associations to followers’ inferior occupational well-being in comparison to transformational leadership; it seems that LMX could buffer follower stress better than transformational leadership. However, as these findings were based on same-source information, they should be evaluated with caution. Previous research has also supported the link between LMX and followers’ occupational well-being, namely, burnout and work engagement (Ellis et al., 2019). However, although the suggested link between the leader’s resources to perform in the leadership role and its consequences for followers’ occupational well-being is theoretically grounded, its empirical verification lies outside the scope of the current study.

### Research Questions

In this study, we aimed to answer four focal questions. First, in order to gain a wider understanding of the various reasons behind leader emergence, we wanted to explore what kinds of factors leaders themselves put forward as having affected their leader role occupancy. Thus, the first research question (RQ 1) was a qualitative investigation of the kinds of reasons that could be identified from leaders’ descriptions of the reasons behind their leader role occupancy. This qualitative approach enabled us to identify the diversity of leader emergence in real world surroundings, as instead of relying on artificial simulations, qualitative research was used to analyze the actual expressions that individuals used in real contexts (Flick, 2014).

Secondly, to assess the role of individual factors and how they are related to the description of reasons for leader role occupancy, we combined quantitative analysis with the aforementioned qualitative descriptions of the reasons for leader role occupancy The qualitative data was quantitized for further analysis (data conversion; see, e.g., Teddlie and Tashakkori, 2009) to enable methodological triangulation. By integrating both qualitative and quantitative data sources (Teddlie and Tashakkori, 2009), we aimed to leverage the strengths of both methodological approaches: we strived to accurately measure and operationalize the constructs of our interest while simultaneously examining the leaders’ experiences in a way that would capture their original, real-life context (Castro et al., 2010). Using statistical analyses, we examined whether leaders’ personal motivation to lead (conceptualized as Affective-Identity MTL, Non-calculative MTL, or Social-Normative MTL) predicted their reasons for leader role occupancy (RQ2).

Finally, to bring together the various reasons behind leader role occupancy and the construction of sustainable leader careers, we explored whether leaders’ different reasons for their leader role occupancy associated with two career sustainability indicators, *health* and *productivity* (De Vos et al., 2020). More specifically, we explored whether the reasons behind leader role occupancy were related to the leader’s health (conceptualized as work engagement and burnout; RQ3) and performance as a leader (conceptualized as followers’ work engagement and burnout; RQ4). To benefit from methodological triangulation and deepen the understanding of different reasons to leader role occupancy and their associations with career sustainability, we will aim to interpret the quantitative findings in the light of the themes identified from the qualitative analysis (integration through data transformation; Fetters et al., 2013).

## Materials and Methods

### Data Collection and Participants

We used multiple sources of data collection to gather data that would be representative of the highly educated leader population in Finland in various fields and industries. An electronic survey was sent via trade unions to gather data that would be representative of different sectors, as the trade unions are organized by industry in Finland, and most Finnish employees are unionized (Ahtiainen, 2015). The survey was composed of carefully chosen self-evaluation inventories and open-ended questions to collect both quantitative and qualitative data simultaneously. In the first phase, a link to the survey was sent to the members of four trade unions: the Finnish Union of University Professors, Finnish Union of University Researchers and Teachers, Finnish Business School Graduates, and Academic Architects and Engineers in Finland. An additional round of data collection was conducted to increase the number of participants. In this additional data collection phase, we used three different data collection sources. One of these was another trade union: the Confederation of Unions for Professional and Managerial Staff in Finland (Akava), which is a confederation of trade unions for those with a university degree or other higher education. This trade union was used to target the survey at social and health care sector leaders. Participants were also recruited from an executive MBA (EMBA) program, and finally, psychology students volunteered to recruit highly educated leaders from among their acquaintances. Altogether, the data collection was conducted during a 6 month period. Participants from the EMBA program and the leaders recruited by students represented various sectors (e.g., the service sector, media and marketing, finance, and insurance, industry) and they were combined for the purposes of this study to constitute one data source. A detailed description of the data collection and response rates for each data source is presented in Auvinen et al. (2020).

#### Leaders

This study focused on leaders who answered the open-ended question about leader role occupancy (*n* = 1,219). Of these 1,219 leaders, 132 had to be omitted from the study as their answer consisted in practice of only a failure or refusal to answer (e.g., “???” or “N/A”), resulting in a study population of 1,087 leaders. Of these participants, 56 gave an answer that, could not be understood in this context (e.g., “Look at my response to the previous open-ended question”). That left a total of 1,031 individual descriptions that could be analyzed. Of the participants, 375 (36%) were professors, 99 (9%) university teachers and researchers, 186 (18%) business sector leaders, 100 (10%) academic engineers, 110 (11%) social and health care sector leaders, and 161 (16%) were “other” highly educated leaders; that is, they had been recruited by psychology students or had an eMBA degree. 51% of the studied leaders were women, the mean age in the sample was 51.4 (*SD* = 8.8) years, and the mean length of past leadership experience was 12.9 years (*SD* = 8.5). Ninety six percentages were working full-time and 99% had a permanent job. Every leader who participated was asked to recruit their followers to the survey anonymously. Leaders were given the information about data privacy and they were requested to send a link to the survey to their followers. The surveys for leaders and followers were identical regarding the focal measures related to the research project, but followers’ survey also included measures to assess their leader’s behaviors and performance.

#### Followers

To assess followers’ experiences, we used hierarchical leader-follower data. Of the leaders who participated in the study, 233 were willing to recruit their followers to participate, and they forwarded an electronic link to their subordinates. The responses were collected in such a way that they were visible only to the researchers, via an electronic survey tool. The data from the leaders and followers were matched by means of code identifiers: followers’ ratings were combined with the data of their closest supervisor who had recruited them to participate in the study. The hierarchical sample included altogether 987 followers of the aforementioned 233 leaders. The number of follower participants per leader ranged between 1 and 14 (*M* = 4.2). Of the followers studied here, 67% were women, the majority (58%) were aged 31–50 years, and the average duration of the relationship with the supervisor who had sent the invitation to take part in the survey was 3.5 years (*SD* = 3.4).

### Measures

#### Reasons Behind Leader Role Occupancy

We used one open-ended question to capture the variety of personal reasons behind leaders’ current leader role occupancy: “What factors have contributed to your having your present position as a leader?” This question was followed by an empty space in which the leaders could type their answers, and there was no word limit. The respondents were thus able to describe as many factors as they chose as having affected their leader role occupancy.

#### Motivation to Lead

We measured leaders’ leadership motivation by using the Finnish translation of the Motivation to Lead Questionnaire (Chan and Drasgow, 2001). A shortened nine-item version of the scale (MTL-9) was used, which has been found to provide a good factor structure validity (see Auvinen et al., 2020). Each sub-dimension of the MTL-9 includes three items; e.g., “I am the type of person who likes to be in charge of others” (Affective-Identity MTL), “It is appropriate for people to accept leadership roles or positions when they are asked” (Social-Normative MTL), and “I never expect to get more privileges if I agree to lead a group” (Non-calculative MTL). All items were answered on a 5-point Likert scale (1 = totally disagree—5 = totally agree), higher scores indicating higher motivation. All of the scale items are available on request from the first author. The Cronbach’s alphas for leaders’ MTL dimensions were 0.92, 0.89, and 0.74, respectively.

#### Work Engagement

Both leaders’ and followers’ work engagement were measured with the nine-item version of the Utrecht Work Engagement Scale (Schaufeli et al., 2002; Seppälä et al., 2009). The scale was used to measure three dimensions of work engagement: vigor, dedication and absorption. Each dimension was measured with three items; e.g., “At work, I feel that I am bursting with energy” for vigor, “I am proud of the work I do” for dedication, and “I get carried away when I’m working” for absorption. Items were answered on a frequency-based scale ranging from 1 to 7 (1 = never, 7 = daily), higher scores indicating more frequent experiences of work engagement. In the leader data, the Cronbach’s alphas for work engagement dimensions were 0.87, 0.89, and 0.83 for vigor, dedication and absorption, respectively. In the follower data, the comparable figures were 0.87, 0.88, and 0.85.

#### Burnout

Both leaders’ and followers’ burnout were measured with the nine-item Bergen Burnout Inventory (BBI-9), which has shown time- and sample-invariant factor structure (Salmela-Aro et al., 2011; see also Feldt et al., 2014). It captures three dimensions of burnout: exhaustion (3 items; e.g., “I am snowed under with work”), cynicism (3 items; e.g., “I feel dispirited at work and I think of leaving my job”) and inadequacy (3 items; e.g., “My expectations for my job and my performance have reduced”). All of the items were answered on a 6-point Likert-type scale ranging from 1 (totally disagree) to 6 (totally agree), higher scores indicating higher burnout. In the leader data, the Cronbach’s alphas for the dimensions of burnout were 0.75, 0.83, and 0.79 for exhaustion, cynicism and inadequacy, respectively, and for the follower data, the comparable figures were 0.72, 0.81, and 0.77.

#### Control Variables

Of leaders’ demographic factors, we investigated age (continuous; in years), gender (dichotomous; 0 = male, 1 = female), past leadership experience (continuous; in years) and occupational sector (membership for each studied sector as a dummy-variable) in relation to focal outcomes. Dummy variables (0 = not a member, 1 = member) were used to following occupational sectors: professors, university teachers, and academics, business sector leaders, academic engineers, social and health care sector leaders and eMBA alumni, and others. These demographic factors were chosen based on their previously found significance in leader role occupancy: gender differences regarding leadership still exist (Kossek et al., 2017) and, on the basis of earlier results from the current data (Auvinen et al., 2020), leaders of different ages and from different occupational sectors differ in their leadership motivation. Those demographic factors that were related to the leader outcomes studied here were controlled for in further analyses.

For the analysis of the followers’ data, the following demographic factors were examined: the follower’s gender (dichotomous; 1 = female, 2 = male), age (categorical; under 20 years, 21–30, 31–40, 41–50, 51–60, over 61 years) and duration of the relationship with closest supervisor (who provided the research request; continuous in years). Correlations and descriptive information about the study variables for the follower data is available from the first author on request. Those demographic variables that associated with followers’ occupational well-being were controlled for in subsequent analyses.

### Analysis

#### Qualitative Analysis: Categorization of Factors Affecting Leader Role Occupancy

We used the merged concurrent nested approach (Tashakkori and Creswell, 2007; Teddlie and Tashakkori, 2009; Castro et al., 2010), which enabled us to achieve a more comprehensive understanding of the varying nature of the reasons why leaders occupy their leadership role. By choosing this approach, we were able to overcome some of the common shortcomings of the mixed-method research design and complement the existing literature on leader emergence. The possibility of simultaneously gathering qualitative and quantitative data enabled us to tackle the general limitation of the *sequential* temporal order of data collection (Bryman, 2007). In concurrent nested approaches, both data sources are collected simultaneously, but greater importance is attached to one type of data over the other (Creswell et al., 2003; Castro et al., 2010). In the present study, we quantitized the reasons the leaders named for occupying their leadership role and we statistically analyzed the associations between these reasons and the indicators of leader career sustainability and personal leader motivation. This approach enabled us to explore how leader role occupancy occurs in the real world; it also allowed us to generalize the findings to a wider population (Fetters et al., 2013). The concurrent approach enabled us to integrate both data sources in an unbiased manner as both were treated as independent entities in the data collection phase, but were brought together for analysis and interpretation (Bryman, 2007). To analyze RQ1, the first author read the leaders’ open-ended answers using an inductive approach in order to identify common themes in the answers. These themes were then grouped together around similar content, resulting in 26 themes (e.g., “Leadership experience,” “Personal characteristics,” “Organizational factors”). After this, two independent coders (psychology students who were trained to do the coding as a part of their studies) read the open-ended answers and coded them according the different themes. Krippendorff’s alpha (Krippendorff, 2004a, b) was calculated for each of the 26 themes based on the work of the two independent coders. The mean level of Krippendorff’s alpha was 0.52 in the whole data, ranging from −0.02 to 0.77. A negative Krippendorff’s alpha indicates a skewness in the variable (Krippendorff, 2004b) and in the present data it concerned two categories that were quite minor in frequency (“Entrepreneurial motives” and “Strive to coach”). After the examination of Krippendorff’s alphas, the first author did a second, blind reading of all the responses and, working on the codings of the two psychology students, came up with a final classification of each response into the 26 themes. The 26 themes were then reviewed to find out whether they form a hierarchical structure and represent a broader phenomenon (Braun and Clarke, 2006, 2012). After the first inductive reading, we were able to identify elements in the data that reflect the key concepts of leader emergence (agency) and sustainable careers (person, context, time), so we took a deductive, theory-driven approach (Braun and Clarke, 2012) to the data to reduce the original number of themes (26), and re-classified the original themes into four new categories: agency vs. non-agency, and the three aforementioned components of a sustainable career.

To answer the criticism of overemphasizing the role of individual agency in career construction (e.g., Inkson et al., 2012), we paid particular attention to differentiating agency from non-agency. A key distinction between agentic vs. non-agentic factors behind leader role occupancy related to whether or not the leaders’ answers included an element of active pursuit or striving toward a specifically leader career. For example, one of the original, first-round content categories, “Leadership experience,” was coded as agentic, as these answers reflected individual striving for a leader position by acquiring experience that would be relevant to the position. In contrast, the category “Experience” was coded as non-agentic, as the answers in this category lacked the active pursuit of a *leader* career and reflected experience other than that related to leadership.

**FIGURE 1:**
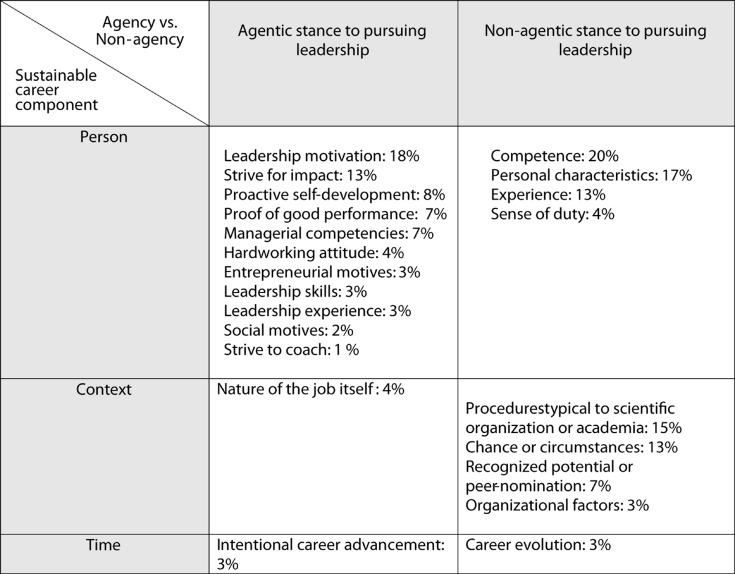
3 × 2 matrix of identified themes in relation to sustainable career components and agentic or non-agentic stance towards leadership. The prevalence of the original theme (one or more mentioning) among all responses presented as %.

Of the 26 content categories that were originally identified in the data, 22 could be identified in the theory-driven analysis focused on the sustainable career components (person, context, time) and agency vs. non-agency. Three of the four categories that were omitted from the final classification lacked the element of agency—non-agency (“Other factors,” “Collaborative skills” and “International experience”) and the fourth one, “Lobbying” (<1% of all responses), was very marginal. Specimen answers and the final theory-driven coding that was used in subsequent statistical analyses are presented in Table 1. The theory-driven classification combining sustainable career components and the level of agency resulted in a 3 × 2 (*Person—Context—Time* × *Agency—Non-agency*) matrix, which was discussed and agreed among the research group and is presented in Figure 1.

**TABLE 1:** The coding procedure for empirical data: Citations, themes, their descriptions and theory-driven codings.

Citation	Theme label	Interpretation	Theory-driven coding
“Previous leadership experience and evidence of good performance, —.” “Previous success as a team leader”	Leadership experience	Positive experiences of prior leader positions and their importance	Person; Agentic
“My own desire to make an impact, to develop and drive things forward”	Strive for impact	Striving to have an impact and develop the present situation	
“I’ve applied for these roles/positions myself” “My own interest in these positions.”	Leadership motivation	Personal interest in leading others, one’s own motivation for positions of leadership	
“(It was) My own goal and hard work to achieve that goal —”	Hardworking attitude	Descriptions of determination and stamina in relation to work	

”— the desire to change to more responsible and more demanding duties.” “The desire to move on from my earlier job. I’m working in this supervisory position because I wanted a job with challenges!”	Nature of the job itself	Highlighting the occupation or the work, increasing or maintaining its meaningfulness	Context; Agentic
“Job description.”			

“My personal characteristics, I believe I am seen as an approachable and positive person.—” “I’m a fit, experienced and reliable person for the job.” “My personality and reputation. — My desire to solve matters sensibly. Courage.” “A sense of responsibility and duty. My desire to do my part for the administration.” “I couldn’t say ‘no’.”	Personal characteristics Sense of duty	Respondent’s perception of his/her own qualities that are suitable for leader Accepting a leader role out of a sense of duty and responsibility.	Person; Non-agentic

“Chance plays a role. I’ve gradually drifted towards leader positions. —” “Everyone takes it in turn.” “There is no one else to appoint as leader.” “The need for someone else to step in because of retirement, organizational changes.” “This is a small academic subject and I’m the only one with a professorship.” “Leading research projects is a ‘natural’ part of being a professor–.” “Leading a research project is naturally something you have to do if you do research and want to organize the work of your colleagues and postgraduate students, —.”	Chance or circumstance Organizational factors Procedures typical of scientific organizations or academia	Prevailing circumstances that were described as outside of the respondent’s active control Descriptions that highlighted the needs of the organization, stemming from organizational restructuring, the size of the unit/department, filling a void, etc. Factors that are typical of academia and the scientific community and affect leader role occupancy: acquiring research funding, leading one’s own research projects, status (e.g., a professorship)	Context; Non-agentic

“The desire to move forward career-wise” “My personal interest and desire to advance in my career”	Intentional career advancement	Descriptions that reflected an intentional advancement of one’s own career up the hierarchy or towards a better position or status	Time; Agentic

“It’s a natural progression in my career and the attendant increase in responsibilities.” “A long career and its ‘normal or typical’ progression.”	Career evolution	Descriptionsthat highlighted career progression as “evolution” and a leader position as an inevitable result of long tenure.	Time; Non-agentic

#### Integrative Analysis: How Quantitized Reasons for Leader Role Occupancy Associates With Demographics, Leadership Motivation and Sustainable Career Indicators

After theory-driven categorization of themes, the qualitative data was quantitized for further analysis (Teddlie and Tashakkori, 2009; Fetters et al., 2013). We used the theory-driven categories of sustainable career components and the coded levels of agency as the starting point for the data conversion and coding. Instead of calculating the exact number of themes in each of the theory-driven categories for every respondent, we used dichotomous coding for each category. This decision was based on our interest in studying whether or not a leader had mentioned each of the themes, not in studying the exact number or distribution of themes within each category. The data conversion procedure resulted in one dichotomous variable (0 = leader had not mentioned this reason, 1 = leader had mentioned one or more reasons in this category) for each of the categories, that were: *person-related, context-related, agentic*, and *non-agentic* reasons behind leader role occupancy.

All of the statistical analyses were performed in SPSS (version 24). The relationships between study variables were investigated with correlation coefficients (Pearson’s *r* and Spearman’s *rho*; available on request from the first author), cross-tabulation, Chi square tests and analysis of variance (ANOVA) to determine the control variables for subsequent analysis (Tables 2, 3). Cross-tabulation with Chi square tests was performed to determine whether occupational background and gender associated with the aforementioned four reason categories. For continuous variables (age, past leadership experience), a similar investigation was performed with ANOVA. Those demographic variables that were found to associate with reason categories were controlled for in subsequent analyses.

**TABLE 2:** Differing demographic factors (occupational group, gender) according to reasons for leader role occupancy.

	Person-related reasons			Context-related reasons			Agentic reasons			Non-agentic reasons		
	None % (adj. res.)	One or more % (adj. res.)	χ^2^ *(df)*	None % (adj. res.)	One or more % (adj. res.)	χ^2^ *(df)*	None % (adj. res.)	One or more % (adj. res.)	χ^2^ *(df)*	None % (adj. res.)	One or more % (adj. res.)	χ^2^ *(df)*
Occupational group			133.07 (5)***			141.89 (5)***			143.64 (5)***			29.20 (5)***
Professors (*n* = 375)	43.5 (10.3)	56.5 (−10.3)		39.0 (−9.9)	61.0 (9.9)		62.5 (9.9)	37.5 (−9.9)		21.5 (−3.9)	78.5 (3.9)	
University teachers and academics (*n* = 99)	34.3 (2.1)	65.7 (−2.1)		43.1 (−3.2)	56.9 (3.2)		55.9 (2.7)	44.1 (−2.7)		22.5 (−1.4)	77.5 (1.4)	
Business sector (*n* = 186)	11.3 (−5.1)	88.7 (5.1)		77.8 (6.1)	22.2 (−6.1)		32.0 (−3.5)	68.0 (3.5)		28.9 (0.2)	71.1 (−0.2)	
Academic engineers (*n* = 100)	15.6 (−2.6)	84.4 (2.6)		81.7 (5.2)	18.3 (−5.2)		38.5 (−1.0)	61.5 (1.0)		39.4 (2.7)	60.6 (−2.7)	
Social and health care (*n* = 110)	16.8 (−2.3)	83.2 (2.3)		61.9 (0.9)	38.1 (−0.9)		28.3 (−3.4)	71.7 (3.4)		29.2 (0.2)	70.8 (−0.2)	
EMBA alumni’s and other (*n* = 161)	6.7 (−6.1)	93.3 (6.1)		73.2 (4.2)	26.8 (−4.2)		14.6 (−8.0)	85.4 (8.0)		40.2 (3.7)	59.8 (−3.7)	
Gender			11.15 (1)***			0.00 (1) *ns*			18.12 (1)***			0.43 (1) *ns*
Female	21.3 (−3.3)	78.7 (3.3)		58.2 (0.0)	41.8 (0.0)		36.8 (−4.3)	63.2 (4.3)		27.4 (−0.7)	72.6 (0.7)	
Male	30.1 (3.3)	69.9 (−3.3)		58.1 (0.0)	41.9 (0.0)		49.5 (4.3)	50.5 (−4.3)		29.2 (0.7)	70.8 (−0.7)	

***p ≤ 0.001, adj. res. = adjusted residuals, ± 2 considered as atypical.

**TABLE 3:** Differing demographic factors (age, past leadership experience) according to reasons for leader role occupancy (ANOVA).

	Person-related reasons			Context-related reasons			Agentic reasons			Non-agentic reasons		
	None *M (SD*)	One or more *M (SD*)	*F (df)*	None *M (SD*)	One or more *M (SD*)	*F (df)*	None *M (SD*)	One or more *M (SD*)	*F (df)*	None *M (SD*)	One or more *M (SD*)	*F (df)*
Age	54.4 (8.3)	50.6 (8.8)	38.89 (1)***	50.3 (8.8)	53.2 (8.6)	28.66 (1)***	54.0 (8.1)	49.7 (8.9)	65.60 (1)***	50.5 (9.0)	51.9 (8.7)	5.56 (1)*
Past leadership experience	12.3 (7.9)	13.2 (8.6)	2.46 (1) *ns*	13.2 (8.6)	12.7 (8.3)	1.08 (1) *ns*	12.4 (8.3)	13.4 (8.6)	3.19 (1) *ns*	13.7 (8.9)	12.7 (8.3)	3.44 (1) *ns*

ns, non-significant; *p ≤ 0.05; ***p ≤ 0.001.

Logistic regression analyses were performed to examine whether a leader’s personal leadership motivation predicted a specific reason category; that is, the mentioning (no/yes) of a specific reason for leader role occupancy. The logistic regression model was estimated independently for each reason category (person- and context-related, agentic and non-agentic reasons). Based on the investigation of the associations between demographic factors and reasons for leader role occupancy, professors were set as a reference group, as they differed from the other occupational groups and were also older than other leaders in the data. To investigate which reasons for leader role occupancy the leader had mentioned and whether their reasons differed in relation to both the leader’s and followers’ occupational well-being, we used analysis of covariance (ANCOVA).

## Results

### Descriptions of Reasons Behind Leader Role Occupancy

The complete list of themes of reasons behind leader role occupancy and descriptions of their content, together with the theory-driven categorization is provided in Table 1. The original themes that were recognized in the open-ended question responses were placed in a 3 × 2 matrix (Figure 1), which was based on the sustainable career components in relation to personal agency. Here, all the original text scripts were analyzed based on whether or not the leaders’ responses reflected personal agency in their pursuit of a career as a leader. For example, responses reflecting the sustainable career component, “Context,” were evaluated to determine if they reflected an active or a passive stance for pursuing a career as a leader. When the respondents described that they were “working in this supervisory position because (they) wanted a job with challenges!,” they mentioned a job-related reason, which emphasized the active role they played. This response highlighted the work itself as the main reason for leader role occupancy, which is a context-related factor according to the model by De Vos et al. (2020) with active, agentic elements. Therefore, it was categorized as Context-Agentic. In contrast, the responses that included context-related themes but reflected a more passive stance (e.g., “Chance plays a role. I’ve gradually drifted toward leader positions”), were categorized as Context-Non-agentic. Each theme was evaluated in a similar manner. To ensure the validity of constructing this matrix, the theme categorizations and the original text scripts were analyzed. This was done by the first author. All the authors discussed and agreed upon the procedure and the final classifications.

As shown in Table 1 and Figure 1, the reasons that leaders mentioned for their leader role occupancy could be classified in terms of sustainable career components (person, context, and time) and the presence of agency (agentic vs. non-agentic). For time-related reasons for leader role occupancy, two mutually exclusive categories appeared in the data: “Intentional career advancement,” which reflected an agentic stance, and “Career evolution,” which reflected a non-agentic stance. Altogether, only 6% of the leaders mentioned a time-related factor (either agentic or non-agentic) that had affected their leader role occupancy. As these categories differed from the others because of their mutually exclusive nature (respondents mentioned either an agentic or a non-agentic stance in relation to time) and limited variation, they were omitted from the subsequent statistical analyses.

Of the sustainable career components, the respondents mentioned more person-related reasons (74%) than context-related reasons (42%) for leader role occupancy. Of the person-related reasons, “Competence” (descriptions of individual qualifications, knowledge, and general or field-specific competencies) was mentioned most often, in 20% of all cases. This was followed by “Leadership motivation,” which reflected the respondent’s personal interest in leading others (18%), and “Personal characteristics” (related to personality and other personal features that are considered suitable for a leader; 17%). Other person-related reasons were “Striving for impact” (the desire to have an impact and to develop the present situation; 13%) and “Experience” (having general experience of life and work experience, a long work history or job tenure; 13%). Person-related reasons pertaining to leadership were mentioned less often: “Managerial competencies” (related to so-called management skills and competencies, e.g., the ability to organize, make decisions, and direct administrative procedures) was mentioned in only 7% of all of the responses given. “Leadership experience” and “Leadership skills” (focusing on skills and competencies related to people management and leadership in contrast to task management, e.g., the ability to communicate vision to followers) were both mentioned in only 3% of the total responses.

Of the context-related reasons, the most typical categories were “Procedures typical of scientific organizations or academia” (15% of all reasons mentioned), “Chance or circumstance” (13%), “Recognized potential or peer nomination” (7%), “Nature of the job itself” (4%), and “Organizational factors” (3%). The category “Procedures typical of scientific organizations or academia” described situations where the position of leader was an automatic consequence of the responsibilities associated with conducting research (e.g., leading one’s own research group) or having an academic position (e.g., a professorship). The category “Chance or circumstances” included answers that highlighted circumstances outside of the individual’s control, such as chance, being the only one who could be appointed leader, or the result of job rotation. This differed from the category “Organizational factors,” in which respondents described the specific needs of the organization and organizational changes that had affected their current leader role occupancy. The category “Recognized potential or peer nomination” included answers that highlighted occupying a leader role because of having been recommended by one’s own supervisor or colleagues, or resulting from peer nomination. The answers in the category “Nature of the job itself” reflected factors related to the content of the work, the need to keep one’s job or increase its meaningfulness, or one’s job satisfaction.

Altogether, non-agentic reasons for leader role occupancy were mentioned more often than agentic reasons: one or more non-agentic reasons were mentioned in 92% of the responses, whereas one or more agentic reasons for leader role occupancy were mentioned in 73%. A complete list of all the identified categories, example answers, and descriptions of the categories is available from the first author on request.

### Results of the Descriptive Analysis: Relationships Between Study Variables

The differing demographics among the reason categories are presented in Tables 2, 3. Taken together, women mentioned more often than men many person-related and agentic reasons for their leader role occupancy. With regard to occupational background, leaders in academic settings tended to express more context- and non-agentic factors that had affected their current leader role occupancy. It was more common for those working in the business sector or with some kind of formal training in leadership to express person-related and agentic factors as reasons for working in a leader position. Older leaders typically mentioned many context-related reasons and no person-related reasons for their leader role occupancy, and younger leaders mentioned more agentic reasons. Past leadership experience was not related to the reasons studied for leader role occupancy. These differing demographic factors were set as covariates in subsequent analyses.

### Personal Leadership Motivation and Leader Role Occupancy

Our second research question was concerned with whether leaders’ personal motivation to lead predicted their reasons for leader role occupancy. The results of logistic regression analyses are presented in Tables 4, 5. Based on the descriptive quantitative analysis, professors were set as a reference group in all the logistic regression analyses as they were older than the other leaders in the study and they differed from leaders with other occupational backgrounds in the reasons for their leader role occupancy in every studied category. Taken together, various factors predicted the mention of person-related and agentic reasons for leader role occupancy, but only occupational background was associated with mentioning reasons that stemmed from contextual factors or reflected a non-agentic stance toward leadership.

**TABLE 4:** Predictors of person- and context-related reasons for current leader role occupancy (logistic regression analysis).

Person-related reasons for leader role occupancy							Context-related reasons for leader role occupancy						
					95% CI for							95% CI for	
					Exp(B)							Exp(B)	
Predictor	β	S.E	Wald’s	Exp(β)	Lower	Upper	Predictor	β	S.E	Wald’s	Exp(β)	Lower	Upper
		β	χ^2^	(OR)					β	χ^2^	(OR)		
Affective-Identity MTL	0.39***	0.12	11.41	1.48	1.18	1.87	Affective-Identity MTL	−0.20*	0.10	3.81	0.82	0.67	1.00
Non-calculative MTL	0.18^†^	0.10	3.40	1.20	0.99	1.45	Non-calculative MTL	−0.01*ns*	0.08	0.01	0.99	0.84	1.17
Social-Normative MTL	0.07*ns*	0.11	0.44	1.07	0.87	1.32	Social-Normative MTL	−0.07*ns*	0.09	0.61	0.93	0.78	1.11
Gender	0.38*	0.17	4.82	1.47	1.04	2.06							
Age	−0.01*ns*	0.01	0.71	0.99	0.97	1.01	Age	0.01*ns*	0.01	0.40	1.01	0.99	1.02
University teachers and researchers	0.24*ns*	0.26	0.86	1.27	0.76	2.12	University teachers and researchers	−0.18*ns*	0.24	0.55	0.84	0.53	1.34
Business sector	1.49***	0.28	28.75	4.44	2.58	7.67	Business sector	−1.62***	0.23	51.20	0.20	0.13	0.31
Academic engineers	1.39***	0.32	18.70	4.01	2.14	7.53	Academic engineers	−1.80***	0.29	39.40	0.17	0.10	0.29
Social and health care sector	1.25***	0.32	14.85	3.48	1.85	6.57	Social and health care sector	−0.94***	0.24	15.63	0.39	0.24	0.62
EMBA alumnis and other	1.97***	0.35	32.40	7.16	3.64	14.11	EMBA alumnis and other	−1.35***	0.23	34.08	0.26	0.17	0.41
Constant	5.32***	0.96	3.45	204.22			Constant	−4.86***	0.84	33.36	0.01		

Test				*X ^2^*	R^2^	%	Test				*X ^2^*	R^2^	%

Goodness-of-fit test							Goodness-of-fit test						
Hosmer and Lemeshow				6.65*ns*			Hosmer and Lemeshow				6.03*ns*		
Nagelkerke R^2^					0.20		Nagelkerke R^2^					0.18	
Cox and Snell R^2^					0.13		Cox and Snell R^2^					0.13	
Overall presentage						75.80	Overall presentage						67.90
Omnibus tests of model coefficients				142.55***			Omnibus tests of model coefficients				14.09***		

ns, non-significant; ^†^p ≤ 0.07; *p ≤ 0.05; ***p ≤ 0.001. All df’s for beta coefficients = 1. For person-related reasons, df = 8 for Hosmer-Lemeshow and df = 10 for Omnibus test. For context-related reasons, df = 8 for Hosmer-Lemeshow and df = 9 for Omnibus test.

**TABLE 5:** Predictors of agentic and non-agentic reasons for current leader role occupancy (logistic regression analysis).

Agentic reasons for leader role occupancy							Non-agentic reasons for leader role occupancy						
					95% CI for							95% CI for	
					Exp(B)							Exp(B)	
Predictor	β	S.E	Wald’s	Exp(β)	Lower	Upper	Predictor	β	S.E	Wald’s	Exp(β)	Lower	Upper
		β	χ^2^	(OR)					β	χ^2^	(OR)		
Affective-Identity MTL	0.62***	0.11	33.16	1.85	1.50	2.29	Affective-identity MTL	−0.31**	0.11	8.10	0.74	0.59	0.91
Non-calculative MTL	0.04*ns*	0.09	0.25	1.04	0.88	1.24	Non-calculative MTL	0.09*ns*	0.09	1.08	1.10	0.92	1.30
Social-Normative MTL	−0.17*ns*	0.09	3.15	0.85	0.70	1.02	Social-Normative MTL	1.00*ns*	1.00	1.09	1.10	0.92	1.33
Gender	0.37*	0.16	5.57	1.44	1.06	1.96							
Age	−0.02**	0.01	6.10	0.98	0.96	1.00							
University teachers and researchers	−0.06*ns*	0.25	0.05	0.94	0.58	1.54	University teachers and researchers	−0.09*ns*	0.28	1.00	0.92	0.53	1.58
Business sector	0.70***	0.21	1.70	2.02	1.33	3.07	Business sector	−0.26*ns*	0.22	1.45	0.77	0.51	1.18
Academic engineers	0.65**	0.25	6.74	1.91	1.17	3.12	Academic engineers	−0.75**	0.25	9.48	0.47	0.29	0.76
Social and health care sector	1.14***	0.27	17.55	3.14	1.84	5.35	Social and health care sector	−0.42*ns*	0.25	2.82	0.65	0.40	1.07
EMBA alumnis and other	1.79***	0.27	43.13	5.99	3.51	1.21	EMBA alumnis and other	−0.83***	0.22	15.00	0.44	0.29	0.66
Constant	3.69***	0.86	18.67	4.19			Constant	−0.65*ns*	0.88	0.547	0.52		

Test				*X ^2^*	R^2^	%	Test				*X ^2^*	R^2^	%

Goodness-of-fit test							Goodness-of-fit test						
Hosmer and Lemeshow				5.57*ns*			Hosmer and Lemeshow				2.95*ns*		
Nagelkerke R^2^					0.23		Nagelkerke *R*^2^					0.06	
Cox and Snell R^2^					0.17		Cox and Snell R^2^					0.04	
Overall presentage						67.9	Overall presentage						71.8
Omnibus tests of model coefficients				186.35***			Omnibus tests of model coefficients				39.78***		

ns, non-significant; *p ≤ 0.05; **p ≤ 0.01; ***p ≤ 0.001. All df’s for beta coefficients = 1. For agentic reasons, df = 8 for Hosmer-Lemeshow and df = 10 for Omnibus test. For non-agentic reasons, df = 8 for Hosmer-Lemeshow, and df = 8 for Omnibus test.

Of the controlled demographic variables, gender, age and occupational group predicted the mentioning of person-related reasons (Table 4) and agentic reasons for leader role occupancy (Table 5). For context-related reasons (Table 4) and non-agentic reasons (Table 5), occupational background was the only demographic factor to predict the mentioning of these reasons for leader role occupancy.

Of the different dimensions of leadership motivation, only Affective-Identity MTL associated with the reasons behind leader role occupancy. It increased the probability of naming person-related and agentic reasons (with odds ratios greater than one) and reduced the probability of naming context-related and non-agentic reasons (with odds ratios smaller than one). Non-calculative MTL failed to reach statistical significance when predicting the mentioning of person-related reasons for leader role occupancy, but had an odds ratio greater than one, indicating increased probability of naming these reasons.

### Leader Role Occupancy and Leader’s Health as an Indicator of a Sustainable Career

The results of the ANCOVA analysis investigating RQ3 are presented in Table 6. With regard to the burnout symptoms that we examined, those leaders who had mentioned one or more person-related reasons for their leader role occupancy reported less cynicism and less inadequacy. With regard to the indicators of work engagement, they also reported more vigor compared to those leaders who had not mentioned any person-related reasons for their leader role occupancy. Conversely, those leaders who had mentioned one or more context-related reasons for their leader role occupancy reported more of all of the burnout symptoms (exhaustion, cynicism, and inadequacy) and less vigor compared to those who had not mentioned context-related reasons at all. As for the agentic reasons behind a leader’s current leader role occupancy, those who had mentioned one or more agentic reasons for occupying their current role reported less cynicism and inadequacy and more vigor and dedication than those leaders who had not mentioned any of these reasons. Non-agentic reasons behind leader role occupancy did not associate with leader’s own occupational well-being.

**TABLE 6:** Mean differences in leader’s occupational well-being according to reasons behind leader role occupancy (ANCOVA; leader’s age, occupational background and gender controlled for).

	Reasons mentioned: None *M (SE)*	Reasons mentioned: One or more *M (SE)*	*F*	*R*_*a*_^2^	Partial η^2^
**Person-related reasons**					
Burnout					
Exhaustion	3.23 (0.08)	3.14 (0.04)	1.01*ns*	0.09	<0.01
Cynicism	2.47 (0.08)	2.28 (0.04)	4.57*	0.02	0.01
Inadequacy	2.73 (0.08)	2.50 (0.05)	5.60*	0.02	0.01
Work engagement					
Vigor	5.50 (0.07)	5.71 (0.04)	5.70*	0.05	0.01
Dedication	5.86 (0.07)	5.94 (0.04)	0.867*ns*	0.02	<0.01
Absorption	5.92 (0.07)	5.88 (0.04)	0.32*ns*	0.02	<0.01
**Context-related reasons**					
Burnout					
Exhaustion	3.04 (0.05)	3.32 (0.06)	12.50***	0.10	0.01
Cynicism	2.24 (0.05)	2.45 (0.06)	7.18**	0.02	0.01
Inadequacy	2.46 (0.06)	2.68 (0.06)	6.25**	0.02	0.01
Work engagement					
Vigor	5.74 (0.05)	5.55 (0.06)	6.02**	0.05	0.01
Dedication	5.96 (0.05)	5.88 (0.05)	1.14*ns*	0.02	<0.01
Absorption	5.91 (0.04)	5.87 (0.05)	0.301*ns*	0.02	<0.01
**Agentic reasons**					
Burnout					
Exhaustion	3.22 (0.06)	3.12 (0.05)	1.66*ns*	0.09	<0.01
Cynicism	2.52 (0.06)	2.20 (0.05)	17.18***	0.03	0.02
Inadequacy	2.70 (0.07)	2.45 (0.06)	8.18**	0.03	0.01
Work engagement					
Vigor	5.50 (0.06)	5.77 (0.05)	12.70***	0.06	0.01
Dedication	5.79 (0.06)	6.02 (0.05)	8.80**	0.03	0.01
Absorption	5.83 (0.05)	5.93 (0.04)	2.04*ns*	0.03	<0.01
**Non-agentic reasons**					
Burnout					
Exhaustion	3.09 (0.07)	3.19 (0.04)	1.68*ns*	0.09	<0.01
Cynicism	2.25 (0.07)	2.36(0.04)	1.83*ns*	0.01	<0.01
Inadequacy	2.52 (0.08)	2.57 (0.05)	0.327*ns*	0.02	<0.01
Work engagement					
Vigor	5.69 (0.07)	5.64 (0.04)	0.41*ns*	0.05	<0.01
Dedication	5.98 (0.07)	5.90 (0.04)	1.07*ns*	0.02	<0.01
Absorption	5.89 (0.06)	5.89 (0.04)	0.01*ns*	0.02	<0.01

ns, non-significant; *p ≤ 0.05; **p ≤ 0.01; ***p ≤ 0.001. Mentioning of person-related reasons: none n = 232, one or more n = 687; context-related reasons: none n = 521, one or more n = 398; agentic reasons: none n = 384, one or more n = 535; non-agentic reasons: none n = 256, one or more n = 663. Burnout scores ranged from 1 to 5, work engagement scores ranged from 1 to 7. R_a_^2^ = Adjusted R Square.

### Leader Role Occupancy, and Leader Performance as an Indicator of a Sustainable Career

The results of the ANCOVA analysis for RQ4 are presented in Table 7. If a leader had mentioned one or more person-related reasons for their leader role occupancy, their followers reported less exhaustion and less inadequacy with regard to the burnout symptoms than the followers of leaders who had not mentioned person-related reasons at all. On the other hand, if the leader had mentioned context-related reasons for their leader role occupancy, their followers reported less vigor than those who had not mentioned these reasons. The followers of a leader who had mentioned one or more agentic reason for their current leader role occupancy reported less exhaustion, but followers’ occupational well-being was not related to a leader’s non-agentic reasons for leader role occupancy.

**TABLE 7:** Mean differences in follower-rated occupational well-being according to leader’s reasons behind their leader role occupancy (ANCOVA; controlled variables presented in the Table notes).

	Reasons mentioned: None *M (SE)*	Reasons mentioned: One or more *M (SE)*	*F*	*R*_*a*_^2^	Partial η^2^
**Leader’s reason for leader role occupancy: Person-related**					
Follower burnout					
Exhaustion	3.12 (0.10)	2.81 (0.04)	8.27**	0.02	0.01
Cynicism	2.17 (0.10)	2.17 (0.03)	0.00*ns*	0.01	<0.01
Inadequacy	2.73 (0.11)	2.48 (0.04)	5.79*	0.01	<0.01
Follower work engagement					
Vigor	5.55 (0.10)	5.74(0.04)	3.09*ns*	<0.01	<0.01
Dedication	5.81 (0.11)	5.85 (0.04)	0.084*ns*	0.01	<0.01
Absorption	5.89 (0.10)	5.77 (0.04)	1.08*ns*	<0.01	<0.01
**Leader’s reason for leader role occupancy: Context-related**					
Follower burnout					
Exhaustion	2.84 (0.04)	2.87 (0.06)	0.25*ns*	0.01	<0.01
Cynicism	2.15 (0.04)	2.21 (0.05)	0.99*ns*	0.01	<0.01
Inadequacy	2.52 (0.05)	2.49 (0.06)	0.10*ns*	0.01	<0.01
Follower work engagement					
Vigor	5.79 (0.04)	5.59 (0.06)	7.90**	0.01	0.01
Dedication	5.88 (0.05)	5.78 (0.06)	1.52*ns*	0.01	<0.01
Absorption	5.78 (0.04)	5.80 (0.06)	0.05*ns*	<0.01	<0.01
**Leader’s reason for leader role occupancy: Agentic**					
Follower burnout					
Exhaustion	3.05 (0.07)	2.78 (0.04)	12.44***	0.02	0.01
Cynicism	2.21 (0.06)	2.16 (0.04)	0.46*ns*	0.01	<0.01
Inadequacy	2.63 (0.08)	2.47 (0.04)	3.33†	0.01	<0.01
Follower work engagement					
Vigor	5.63 (0.07)	5.75 (0.04)	2.39*ns*	<0.01	<0.01
Dedication	5.86 (0.07)	5.83 (0.04)	0.11*ns*	0.01	<0.01
Absorption	5.82 (0.07)	5.77 (0.04)	0.38*ns*	<0.01	<0.01
**Leader’s reason for leader role occupancy: Non-agentic**					
Follower burnout					
Exhaustion	2.80 (0.06)	2.87 (0.04)	1.12*ns*	0.01	<0.01
Cynicism	2.16 (0.06)	2.18 (0.04)	0.03*ns*	0.01	<0.01
Inadequacy	2.59 (0.07)	2.47 (0.05)	1.98*ns*	0.01	<0.01
Follower work engagement					
Vigor	5.72 (0.06)	5.71 (0.04)	0.02*ns*	<0.01	<0.01
Dedication	5.79 (0.06)	5.87 (0.04)	1.05*ns*	0.01	<0.01
Absorption	5.72 (0.06)	5.81 (0.04)	1.58*ns*	<0.01	<0.01

ns, non-significant; ^†^p ≤ 0.07; *p ≤ 0.05; **p ≤ 0.01; ***p ≤ 0.001. Followers’ responses per mentioning of person-related reasons: none n = 110, one or more n = 854; context-related reasons: none n = 605, one or more n = 359; agentic reasons: none n = 239, one or more n = 725; non-agentic reasons: none n = 302, one or more n = 662. For burnout, the length of leader-follower relationship was controlled for. For work engagement, followers’ age, gender and the length of leader-follower relationship was controlled for. R_a_^2^ = Adjusted R Square.

## Discussion

We had four specific aims in this study. First, we wanted to explore the variety of reasons that leaders would mention when asked to name the factors behind their leader role occupancy. Secondly, in order to assess how individual factors predict the reasons for leader role occupancy, we used mixed method analysis strategy to examine if leaders’ personal leadership motivation was one such indication. We also combined the different reasons for leader role occupancy with the model of sustainable leader careers, and explored whether different reasons behind occupying a leader role associated with career sustainability indicators. Thus, our third aim was to investigate the reasons for leader role occupancy in relation to leaders’ health. Finally, our fourth aim was to explore the reasons for leader role occupancy in relation to leader performance, that is, productivity. Let us now turn to our findings in more detail.

### Drivers and Drifters—Various Reasons Affecting Leader Role Occupancy

We found that there was substantial variation in the leaders’ descriptions of what had affected their current leader role occupancy, as the preliminary analysis resulted in 26 different themes. After classifying these themes based on the conceptual model of sustainable careers, we were able to identify all the three components of the model (person, context, time) in our data. We could also identify both agentic and non-agentic attitudes toward pursuing a leader role. Four of the original, data-driven categories did not fit the theory-based classification, as they lacked the level of agency or were too vague to be classified according to the components of a sustainable career. One such example was the category of “Other factors,” in which we placed all those responses that did not fit into any other content categories, such as one that cited money.

Of the person-related reasons that were mentioned as having an effect on the respondent’s present position as a leader, the majority reflected an agentic stance toward pursuing the position. Agency in pursuing a leader role was mentioned in 69% of the responses, while 54% of the responses reflected a non-agentic stance toward leadership. Among the agentic person-related reasons, personal leadership motivation was the one most often mentioned for current leader role occupancy (nearly one fifth of the responses). Personal motivation for leader roles acts as natural fuel for leader emergence, and from the sustainable leader career perspective, for an individual a possibility to activate or verify valued personal identities through work, it provides a sense of meaningfulness (Rosso et al., 2010).

Factors related to management or leadership skills or competencies were mentioned strikingly less often than other agentic, person-related reasons (in less than one tenth of the total responses). In addition, reasons that involved being suitable for the job (i.e., having the personal characteristics that are considered appropriate for a leader) was only the third most frequently mentioned reason (preceded by “Competence” and “Leadership motivation”) among all the person-related reasons. This finding is surprising, even alarming, when we consider whether people who work as leaders are actually suitable for their role. Although having personal motivation is important, also task-related skills, competencies, and appropriate personal characteristics are necessary to succeed in the job, and for good leader performance (Van Iddekinge et al., 2009). In addition, the leaders who participated in the study gave nearly as much emphasis to having personal experience that was not related to previous leadership tasks as to having suitable personal characteristics. For example, leaders stated that they were “qualified to do so,” had “acquired experience and competence in such areas (undergraduate education, research, societal interaction) that have provided the prerequisites for academic leadership positions” or generally mentioned “expertise and experience” as a reason for occupying a leader role. This raises questions about the leader’s performance and the quality of his or her leadership: general experience of life and work may be helpful when working as a leader, but is it enough to ensure that the person will perform well in a demanding position with multiple staff- and performance-related responsibilities? It has to be noted, however, that the majority of the leaders in our study came from the academic world, such as universities, so their initial career motivations may have been based more on professionalism than on leading others (see e.g., Chan et al., 2012). This might help to explain the prevalence of these reasons in the data.

Striving to make an impact or to develop the existing situation was mentioned fourth most frequently as a reason for leader role occupancy. This may indicate that despite the falling interest in leader positions (Chudzikowski, 2012; Sutela and Lehto, 2014; Torres, 2014; Crowley-Henry et al., 2019), among those who are working as leaders, the position is still seen as valuable and appreciated, offering the possibility of having an influence on a whole range of different matters, from having an impact on one’s own work community to wider societal issues. Being able to have an impact via one’s work gives one a sense of purposefulness, which is conceptualized as the experience that one’s work serves a broader purpose and something valuable beyond oneself (Steger et al., 2012; Martela and Pessi, 2018). Making a contribution (i.e., serving a broader purpose) is an essential source of meaning (Rosso et al., 2010; Steger et al., 2012; Martela and Pessi, 2018), which is a key element in supporting career sustainability (De Vos et al., 2020). Another theme that was identified in the data as reflecting the importance of contributing as a source of meaning was the desire to coach or mentor others. Leaders told that they had an “interest in guiding people,” “desire to coach and train future leaders and managers” or had an “opportunity to influence and coach subordinates to growth.” Although answers that were classified under this person-related theme were reported by only about one percent of the leaders, it is an important motivational factor that includes at the same time both agentic, individual preferences for leading and more communal preferences for supporting others. In sum, leaders who mentioned person-related reasons for their leader role occupancy can be viewed as active drivers toward their leader position. They are also more likely to draw meaning from multiple sources for their current role as a leader.

The majority of the context-related reasons, as expected, reflected a non-agentic stance to pursuing leadership. The two most often mentioned context-related reasons related to conditions that were outside of one’s personal control. The first related to the well-established practices or patterns within the leader’s organization, which affected their job description and shaped the content of responsibilities within their specific sector. Leaders stated that “it has to be done—(leader positions) fall under the professor’s job description” or that “the post is responsible for the laboratory’s research.” These organizational and/or institutional norms had affected the current leader role occupancy, and they were most prominent among leaders working in academia. The responses of these leaders often included descriptions of adaptation to the current situation and to their work environment. Even though they saw their leadership duties as extra work, beyond their core tasks (such as, for the academics, of conducting research), they did not question or criticize their leader role. Rather, leader responsibilities were accepted as a matter of course. This is not surprising, as research shows that leaders in academia have varying levels of leadership motivation (see Auvinen et al., 2020). These findings raise the question of how these leaders experience meaning in their work: whether it is possible for them to derive any meaning from a leader career if their original career motivation was not leadership-related but something else (e.g., professional motivation; Chan et al., 2012). Taken together, those leaders who emphasized context-related reasons can be seen as drifters in relation to leadership. Ending up as a leader regardless of the fact that they have not themselves set leading others as a personal goal calls for adjustment and adaptation to external circumstances and contextual demands. This may associate with poorer perceived meaning (see also, De Vos et al., 2020), especially if one’s work and the position offer limited openings to other sources of meaningfulness than deriving it from self-actualization or expressing own authentic self (Rosso et al., 2010).

An interesting finding was that women and younger leaders mentioned more person-related and agentic reasons for leader role occupancy than men and older leaders. This may indicate that there is still gender inequality when it comes to occupying leader positions (Kossek et al., 2017). In this study, leaders who were women felt that if they wanted to work as a leader, they had to exert themselves more and demonstrate greater proactivity than their male counterparts. The descriptive statistical analyses of the association between age and occupational background revealed that the professors were significantly older than leaders in other occupational sectors. This is not surprising, as leader positions usually come along later in the course of a university career. However, this association between age and occupational group may partly explain the finding that younger leaders mentioned more person-related and agentic reasons for their leader role occupancy. This would also be explained by the fact that younger leaders are at a different stage in their career from older leaders, and their need to consolidate their position in the labor market is more pronounced, so they are more likely to have more person-related, agentic reasons to get on in their career.

### Intrinsic Leadership Motivation Associated With Leader Role Occupancy

Of the different types of leadership motivation, the intrinsic component, Affective-Identity MTL, had a significant role in predicting all of the four reasons behind leader role occupancy, whereas the other motivation types (selfless, Non-calculative MTL, and extrinsic, Social-Normative MTL) did not. Interesting light can be thrown on the different aspects of leadership motivation when we explore how MTL dimensions predicted mention of the reasons behind leader role occupancy (the odds ratios). All aspects of leadership motivation (intrinsic, extrinsic, and selfless) were related to an increased probability of naming person-related reasons behind leader role occupancy, while with context-related reasons, there was a corresponding decrease in probability. In these two reason categories, different dimensions of MTL operated in parallel, but with opposing effects.

Investigating the mention of agentic and non-agentic reasons reveals the different nature of these leadership motivation components, supporting previous research on MTL dimensionality (Badura et al., 2020). Intrinsic and selfless leadership motivations were related to an increased probability of mentioning agentic reasons for leader role occupancy, whereas extrinsic leadership motivation was related to a decreased probability. On the other hand, both selfless and extrinsic leadership motivation were related to an increased probability of mentioning non-agentic reasons, and intrinsic, identity-like leadership motivation was related to a decreased probability. This indicates that the level of agency differs between MTL dimensions: intrinsic, identity-like leadership motivation clearly associates with the agentic pursuit of leader positions whereas extrinsic leadership motivation associates with a non-agentic stance to leadership. Selfless leadership motivation differs from the intrinsic and extrinsic motivations, as it included both agentic and non-agentic stances to leader positions. These findings support previous research on MTL dimensionality and highlight the importance of studying each MTL dimension separately rather than studying a composite score (Auvinen et al., 2020; Badura et al., 2020). The selfless leadership motivation component, Non-calculative MTL, needs to be studied more extensively, as it differs from the traditional intrinsic –extrinsic classification of motivational constructs.

### Driving or Drifting Toward a Sustainable Leader Career?

Our aim was to connect leader role occupancy to the framework of sustainable leader careers (De Vos et al., 2020) and its key indicators. We examined leaders’ own occupational well-being as an indicator of health and followers’ occupational well-being as an indicator of productivity, that is, leader’s performance as an output of leadership. All of the reasons for leader role occupancy except non-agentic ones were related to both leaders’ own and their followers’ occupational well-being, but the associations were different.

We found that leaders who had more person-related and agentic reasons for leader role occupancy experienced better occupational well-being (less cynicism and inadequacy, and more vigor). Also, leaders who had agentic reasons experienced stronger dedication. These findings suggest that leaders who have personally chosen their leader position, i.e., drivers, thrive in their current role. Their experienced occupational well-being and willingness to dedicate themselves to their work suggests that their chosen position and career provide them with a sense of meaning and offer good person-career fit. This stands in contrast to leaders who gave context-related reasons for occupying their position, who experienced all of the burnout symptoms and less vigor. Leaders who end up in a leader position largely due to external factors (such as the surrounding context; in other words in a position that they have not actively pursued, i.e., they are drifters) may end up being also “victims of circumstance” as regards their well-being. These findings suggest that for drifters, the necessary adaptation to the demands of the surrounding context may be costly, and this will be reflected in their poorer occupational well-being and person-career mismatch. Also, deriving a sense of meaning from their career in the long run is left open to question, as it is likely that for them, the source of meaning will not stem from authenticity and self-realization through their work (Rosso et al., 2010). To support the sustainability of the drifter’s leader career, it is vital to identify what gives the position meaning for them. Do they derive meaning from “unification,” that is, purpose, belongingness and supporting others (Rosso et al., 2010; Martela and Pessi, 2018), and whether acting in a leader position for contextual reasons offers fulfillment of personally important aspects at work (Peterson et al., 2017) other than being in charge of other people and resources?

From the perspective of good leader performance (which we assumed would be reflected in followers’ better occupational well-being), the leader’s reasons behind leader role occupancy were also significant. Person- and context-related and agentic reasons behind leader role occupancy associated with followers’ occupational well-being. The associations for followers’ well-being, however, differed from those of leaders’ well-being: for example, person-related and agentic reasons associated with followers’ exhaustion, but this association was not found among leaders. It could be argued that these findings support the idea of conceptualizing followers’ well-being as an indicator or a consequence of (good) leader performance. If a leader is motivated and has proactively chosen the leader position (reflecting the agentic stance), s/he would probably have sufficient and appropriate resources for leadership (see Auvinen et al., 2020) to perform well in the position. According to resource investment principles (Hobfoll, 1998, 2001, 2011), leader may then be able to invest more resources in vital leader behaviors, such as quality interaction and supporting followers, which could result in better occupational well-being for followers (Harms et al., 2017).

### Theoretical and Practical Contribution

This study contributes to our understanding of the leader emergence process by acknowledging the various reasons that can bring about leader role occupancy. Our mixed-method study with over 1,000 Finnish leaders has shed light on the question of what kinds of different pathways may lead to a leader position. Theoretically, our goal was to offer another, more authentic perspective on leader emergence to complement earlier research, which has mostly been carried out in simulated settings (leaderless group discussions, see e.g., Ensari et al., 2011).

A recent review (Hanna et al., 2021) emphasized that emerging leadership is still largely attributed to individual differences. The findings of this study reiterates the message by Hanna et al. (2021, p. 88) about leader emergence research being in the need of more comprehensive review of “situational or contextual factors that are likely to influence the effects of these individual differences.” To the best of our knowledge, this study was the first to connect the reasons for leader role occupancy to sustainable career indicators. To tackle the overemphasis on the role of agency in career construction (Inkson et al., 2012), more systematic understanding that acknowledges individual, contextual and time-related factors affecting the *process* of leader emergence is needed. In addition, the question of how the process of leader emergence is tied to the construction of sustainable leader careers deserves more attention. Our findings confirm that all the perspectives related to the individual, the context, and time are relevant for leader emergence. It was beyond the scope of this paper to investigate longitudinally the process-like nature of how these different reasons for leader role occupancy may result in endurable leader careers. However, we sought to offer a starting point for such theoretical and empirical developments in the future.

The findings of this study give practitioners an important perspective on supporting sustainable careers. Overall, it was found that the reasons behind a leader’s current leader role occupancy associate with focal career sustainability indicators. It seems likely that those who have actively pursued their leader position, the “drivers,” will be able to build a sustainable leader career with positive consequences in terms of occupational well-being for both themselves and their followers. On the other hand, constructing a sustainable leader career seems less likely for the “drifters,” who have ended up in the leader position largely due to external reasons. This should be seriously taken into account in human resource management/development processes in organizations.

To ensure the well-being and sustainable careers of “drifters,” it is vital to pay attention to two issues: the potential sources of meaning in their work, and the different possible ways of supporting their agency in their current leader role. Regarding the former, it is important to take into account the meanings or needs that are important to individual and how they are fulfilled in one’s current employment (task, position, role; Peterson et al., 2017). Acknowledging the possible gap between what is personally important and its realization in one’s current employment may prompt a move either to establish a better balance between one’s duties and their personal meaning and fulfillment or, alternatively, to make a career shift to a more fulfilling position altogether. The latter, i.e., supporting leader agency, can be done via interventions that help leaders to explore their personal leadership motivation and strengthen the intrinsic component, Affective-Identity MTL (see e.g., Stiehl et al., 2015). This is especially important when designing career practices in those fields where leaders highlighted contextual or non-agentic reasons for leader role occupancy, such as academia and other research-oriented organizations, where primary work-related motivations may stem from sources other than leading others.

### Limitations and Suggestions for Further Research

Our findings were based on leaders’ self-reported qualitative, retrospective descriptions of the reasons behind their current leader role occupancy. So far as we know, this was the first study to utilize this kind of qualitative data to examine leader role occupancy, and therefore the original classification of 26 themes cannot be validated against previous research. We tried to minimize any coder-dependent bias that could have occurred from different ways of interpreting the leaders’ responses by calculating inter-coder reliabilities. In addition, based on those reliabilities, the classification was reviewed and finalized by the first author. It must be noted that the classification of qualitative data reflects the sample characteristics and with a different sample, different themes could have been identified (Braun and Clarke, 2012). To develop a wider understanding of the various reasons that affect leader role occupancy, qualitative data must be collected from diverse samples in future studies. Our qualitative thematic analysis combined inductive reading and theory-driven classification with a realist ontological and epistemological stance (King and Brooks, 2018), as in this data we aimed to identify explicit semantic themes (Braun and Clarke, 2012). Future studies could dig deeper into the question of leader role occupancy and apply a relativist, constructionist stance (King and Brooks, 2018), searching for latent themes to examine how the leader emergence process is constructed in a given organizational–or private–context.

Our data collection enabled leaders to name several reasons that had affected their leader role occupancy. This limits possible inferences about the relational value and importance of each reason for a leader. On the other hand, the question was intentionally left at a quite general level because we wanted to capture leaders’ experiences in the broadest sense. To extend our understanding of the most important factors in the leader emergence process, future studies could focus on the reason individuals prioritized when considering leader role occupancy. This could also help to create a more detailed theoretical model of the leader emergence process and supplement career construction theories, which have been criticized for being too focused on personal factors (see Inkson et al., 2012).

Lastly, we want to point out that due to the cross-sectional study design, no causal inferences can be made about the reasons for leader role occupancy resulting in the creation of sustainable leader careers. To investigate this process more solidly, longitudinal research designs are needed. Future studies should also pay attention to *happiness* indicators of sustainable leader, such as career subjective career success and job satisfaction (De Vos et al., 2020) which were not in the scope for the present study. In examining how to construct sustainable careers, fruitful themes in future research include the role of people’s experience of the meaningfulness of work, the possible differing sources of deriving meaning from work for drivers and drifters, and the costs of adaptation to the context (i.e., working as a leader with low motivational resources or for extrinsic reasons due to the needs of context). These perspectives would both enrich the sustainable careers literature and give insights that would be of practical value in the field of human resources and development.

## Conclusion

In order to build and support sustainable careers, it is important to understand the reasons behind people’s career choices. Acting in a leadership role is one such career choice, and various explanations have been put forward to explain leader emergence. Individual agency in making career choices has been much emphasized, but it is also important to take into account the existence of other, non-agentic or non-person-related factors affecting individual career choices. This study has shown that leader emergence and leader role occupancy can involve many factors in addition to individual agency. In order to support the building of sustainable careers, it is of paramount importance to consider how the different reasons behind career choices are linked to sustainable career indicators. In this study, person-related and agentic reasons for leader role occupancy associated with sustainable career indicators, namely leader’s health (occupational well-being) and the occupational well-being experienced by their followers, which is one way of representing leader performance. The research has yielded important information for practitioners responsible for the development of human resources in organizations, because it has shown that if the reasons for leader role occupancy mainly reflect circumstances or other non-person-related reasons, the experienced meaningfulness of work and person-career fit may remain weak. In this case, it may be necessary to try to strengthen or support the leadership motivation of those in leadership positions, or to shape the job description in such a way that it can also offer the experience of meaningfulness from aspects other than self-realization through a managerial role.

## Data Availability Statement

The datasets presented in this article are not readily available because: the data that support the findings of this study has been collected confidentially and is stored in accordance with GDPR at University of Jyväskylä. The data are not publicly available due to privacy or ethical restrictions. Requests to access the datasets should be directed to EA, elina.m.e.auvinen@jyu.fi.

## Ethics Statement

Ethical review and approval was not required for the study on human participants in accordance with the local legislation and institutional requirements. The patients/participants provided their written informed consent to participate in this study. Written informed consent was obtained from the individual(s) for the publication of any potentially identifiable images or data included in this article.

## Author Contributions

EA, MH, JR, and TF together conceptualized the article and together edited and finalized the article. EA wrote the first version of it and conducted the analysis as described in the manuscript. The submitted version was approved by all authors and all can be held accountable for the content.

## Conflict of Interest

The authors declare that the research was conducted in the absence of any commercial or financial relationships that could be construed as a potential conflict of interest.
